# The American and Colonial Journals: Pathology, Practical Medicine, and Therapeutics

**Published:** 1840-07

**Authors:** 


### II. THE AMERICAN AND COLONIAL JOURNALS.

### PATHOLOGY, PRACTICAL MEDICINE, AND THERAPEUTICS.

*Case of Idiocy from a Blow on the Head.* By S. ANNAN, M.D., Physician to the Baltimore Almshouse Hospital.

E. R., ætat. twenty-five years, admitted May 16th, died August 1st, 1839. This girl was a prostitute; and on Christmas, 1836, was struck on the right parietal bone with a stick by one of her paramours, by which she was knocked down and rendered insensible. She remained in a state of stupor during several hours. For some days she was unable to leave her bed, and she never afterwards had perfect command of her limbs; neither was she able to articulate distinctly, and her intellect was so much impaired that she was regarded as idiotic. When admitted into the Almshouse she could walk a few steps with a tottering gait, but was liable to fall forwards, unless she could catch hold of something wherewith to support herself. On attempting to grasp anything, the motions of the hands and arms were irregular, showing that the muscles were not completely under the command of the will. The motions somewhat resembled those of a person affected with chorea. She appeared to be completely idiotic, and was as easily managed as a child. When she attempted to speak, although with a little attention a number of words could be distinguished, no coherent sentence could be made out. She was frequently seized with what appeared to be a chill. She became paler; the extremities cold; and she whined and wept, and was restless and uneasy for two or three hours. No marked hot stage, however, succeeded. During several weeks before her death, the muscles about the mouth were in a state of constant motion, except when she was asleep. She was attacked with dysentery, which was succeeded by diarrhœa, of which she died.

*Post-mortem* examination twelve hours after death. Brain alone examined. Neither the scalp nor the skull showed any trace of a blow having been inflicted. The arachnoid membrane and pia mater, over the entire surface of both hemispheres of the cerebrum, especially on the upper and lateral parts of the anterior and middle lobes, adhered so firmly to the convolutions, that it was with considerable difficulty they could be stripped off. Over the posterior lobes and base of the cerebrum, the adhesion was not so strong. From the anterior and middle lobes the cineritious matter, in a softened state, came off, adhering to the membranes. The arachnoid was considerably thickened and was opaque. The thickening and opacity were greatest over the superior parts of the middle lobes. The medullary centre of the cerebrum showed a greater number of red points than is natural, and the superficial veins of the ventricles were distended. Three or four ounces of serum were found in the sac of the arachnoid and in the ventricles, and there was a small quantity under the pia mater.

REMARKS. This case would appear to countenance the theory of Foville and Pinel Grandchamp, viz., that the cortical substance is the seat of intelligence. The blow upon the head brought on inflammation of the arachnoid, pia mater, and cortical substance. Extensive adhesions were formed between the membranes and the surface of the convolutions; and softening of the cortical matter took place. There was extensive disorganization of the surface of the cerebrum, while the medullary matter was comparatively uninjured. The mind was a complete wreck; but motion, until she became greatly debilitated, was not very much impaired.

*American Journal of Med. Sciences.* No. xlix. *Nov.* 1839.

*Remarks on the Black Vomit of Yellow Fever.* By F. M. Robertson, M.D., of Augusta, Georgia.

A most destructive epidemic has prevailed in our city since the early part of August. Various opinions were at first entertained as to the nature of the disorder. The symptoms, course, and termination of the disease, in connexion with the post-mortem appearances, leave no doubt as to its being, unequivocally, "yellow fever," or what is styled in Charleston, S. C., "*strangers' fever.*" Some, who still contend that it is not yellow fever, assert that the black matter thrown from the stomach during the life of the patient, and found in it after death, is nothing more than black bile, mixed with the secretions of the stomach, the ordinary production of autumnal bilious fevers.

The late Dr. Physick, I think, was among the first to point out the remarkable difference between the matter of black vomit and the black bile. I repeated his experiments at a post-mortem examination to-day. The subject of the examination had laboured under the usual symptoms of yellow fever, and expired throwing up large quantities of "black vomit." Upon exposing the cavity of the abdomen, the cardiac and pyloric extremities of the stomach were carefully tied, so that nothing could escape from or enter into that viscus: the gall-bladder was secured in the same manner, and both removed from the body. The stomach contained about a pint of black vomit, answering, in every particular, to the description given by different writers upon the subject. The gall-bladder, which was of the natural size, was two thirds full of deep black bile, of nearly the consistence of tar.

I first spread the bile upon a white sheet of paper; it presented the usual dark green tinge, slightly inclining to yellow. The black vomit from the stomach was also treated in the same manner; the colour and appearance were totally different from that of the black bile. The fluid part of the black vomit resembled dirty coffee, while a thick dark sediment, resembling the coffee grounds, was deposited on the paper.

Some of the black bile was next placed in a vessel containing clear water; it was completely soluble, communicating its peculiar colour to the water. The black vomit was next placed in a similar quantity of pure water. After the most violent agitation, the dark flocculi would sink to the bottom of the vessel, no portion being dissolved, and the water returning to its natural appearance. One other test was made; this experiment was tried, as we are informed, by Dr. S. Cooper; I am not at present aware of any other authentic instance. Knowing that the intense bitterness of the bile would be communicated to any substance, however slightly it might be impregnated with it, I resolved to try the different impressions that would be made upon the organs of taste by the two substances. I first tasted the black bile, which was intensely bitter. After washing the mouth thoroughly from the impression produced by the bile, I then tasted the black vomit taken from the stomach. This communicated a slightly sour or subacid taste; with this exception it was entirely insipid—not the slightest traces of bile were discoverable from the taste. This test was repeated by Dr. A. G. Howard, of Charleston, S. C., who assisted in the post-mortem examination, and the same characters were recognized by him.

*American Medical Examiner. Oct.* 19, 1839.

---

*On the Use of Quinine in Yellow Fever.* By the Editors of the Philadelphia Medical Examiner.

Much attention has been directed at New Orleans to the treatment of yellow fever by very large doses of the sulphate of quinine, given at the period of collapse, immediately at the commencement of the disease. This practice, which consists in giving large doses of quinine, say from a scruple to a drachm, has been resorted to, we believe, for two or three years past, with great advantage, to the diminution of the mortality of this disease, which, under ordinary methods of treatment, is singularly fatal.

We do not know at what exact date or at whose suggestion this practice was instituted. Most probably, like other modes of treatment, the idea has often been suggested and successfully acted upon; but it is not long, at least, that the practice has been generally adopted, or that its success has been established upon tolerably certain grounds. In all cases of this kind we are much more inclined to give the credit of a successful mode of treatment, or of the investigation of an obscure pathological problem, to those who first introduce it to the profession, by setting forth the testimony on which it rests in a clear unquestionable light, than to the physician who may have simply conceived the possibility of the connexion, but has let it remain in a useless and impracticable form.

The evidence respecting this new mode of treatment, to which we perceive an allusion in one of the late meetings of the Academy of Medicine of Paris, must be very complete to carry with it entire conviction. It is so easy to mistake the success of practice in epidemics, like yellow fever, which vary every year, and change their character sometimes in the course of a few weeks, that we should not entirely admit that any plan of treatment would be uniformly successful, until it had been tested by the epidemics of several successive years. Still, the experience of a single physician in a single year would furnish just grounds for adopting and testing a practice which may have been unusually successful in his hands, and may promise still better results when tried upon a larger scale.

We have thrown out these remarks more with the hope of eliciting some more definite information from our New Orleans correspondents than we now possess than from any present knowledge we have respecting a mode of practice which, at least, promises more than any hitherto employed.

To put our readers in possession of what facts have come to light on this subject, we extract the following notice from a New Orleans paper:

"*The Use of Quinine in Yellow Fever.*—The Lafayette Gazette mentions the salutary effect in cases of yellow fever, derived from the exhibition of the sulphate of quinine, in very large doses. Several facts coming under our immediate knowledge enable us to corroborate this statement, and to add that we believe the "quinine treatment," as it is termed, to be a most important and incalculably beneficial discovery.

"The manner of employing the quinine in fever cases, which has been followed by such astonishing success, differs altogether from the mode in which that remedy is usually administered. The common practice of physicians has been to give it in small doses during the periods of remission. The new practice is based upon a different theory, and varies essentially from the old. When quinine is taken in large quantities, medical men have observed that it produces but a slight and inconsiderable stimulating effect, which is succeeded within a few hours by a powerful sedative impression, that is generally durable. With this view the medicine is exhibited in one very large dose, of from twenty to sixty or eighty grains, in the very incipiency of the fever, while the morbid action appears to be in process of formation—that is, within six or eight hours immediately after the appearance of the earliest symptoms. It is all important, if we rightly understand the theory of its use, that the quinine should be employed before local irritation or congestion has taken place, or, in other words, while the malady is confined to the nervous system, and the organization is as yet unimpaired. When taken under such circumstances, its first effects are a very slight increase of the febrile symptoms; the pulse perhaps becomes quickened, the respiration more hurried, and the usual consequences of stimuli are present. This condition is, however, but transient, and is promptly followed by corresponding depression. All the more violent symptoms subside; the temperature of the surface is lowered; pain diminished; the pulse is gentle and subdued; the skin is covered with a healthy moisture: in short the chain of morbid associations becomes broken—sleep is superinduced, from which the patient awakes refreshed, and substantially better; and within twenty-four or thirty-six hours is considered in a state of convalescence. The treatment is, of course, not exclusively confined to the employment of quinine, though this is the chief remedial agent. The usual means of obviating tendencies to local irritation must be resorted to. The skilful practitioner will modify his curative measures according to the necessities of the case; cupping, leeching, the warm bath, and local applications, may be used as circumstances call for their employment. The quinine is administered in a single dose; the object of the physician is to bring about the sedative influence of the remedy, before any of the organs, as the head, stomach, &c., become specially affected by the disease. If it should fail to produce the anticipated effect, the case is too far advanced for a second trial, and it must be treated on general pathological principles. Let it, however, be remembered that in thirty or forty cases which have been subjected to this novel curative method, not one has terminated fatally. The action of the quinine has been uniformly most salutary, operating like a charm, and dissipating the symptoms of the malady ere they become concentrated on particular organs.

"We have been an eye-witness of the excellent effects of the quinine treatment in several instances, and can with justice render a tribute to the zeal and talent displayed by some of the members of the profession in the employment of this remedy. We are not aware to whom the merit of the discovery belongs, Those physicians who have paid particular attention to its *modus operandi*, and have employed it in the largest number of cases, are Drs. Hunt, Beattie, Farrell, and Mackay. These gentlemen concur in their views of the theory upon which the treatment is based, as well as in the unexceptionably advantageous results which accrue from its application."

*American Medical Examiner. Oct.* 19, 1839.

---

### *Observations on the Employment of Cimicifuga in the Treatment of Chorea.* By T. S. Kirkbride, M.D.

The Cimicifuga* is well known in many parts of this country under the common name of "black-snake root," and although used for many years in domestic practice, was first brought before the profession as a remedy of value in the treatment of chorea, by Dr. Young, of Chester County, Pennsylvania, in a paper published in the 9th volume of this Journal.

The first two cases that came under our notice, after the publication of Dr. Young's paper, occurred during our residence in the Pennsylvania hospital. One was a lad about ten years of age, and the other a girl of thirteen. In both patients the symptoms were well marked and of some standing. After coming under our care, the treatment consisted of free purging for a few days preparatory to the use of the powdered root of the cimicifuga, in the dose of a teaspoonful three times a day, and frictions over the surface of the body generally. No other remedy was employed, and the improvement which commenced almost with the use of the root, continued till the perfection of the cure, which was in about four weeks in both instances.

The next case we had an opportunity of treating was in private practice, during the summer of 1835. The patient, a delicate girl, ten years of age, had decided symptoms of chorea, but of short duration. After free purging she took a wine-glassful of a decoction of cimicifuga three times a day, and gradually increased the dose. This decoction was made by boiling one ounce of the root in a pint of water for twenty minutes. Her symptoms abated promptly and the cure was complete.

In only one of the cases just noted were the functions of the mind much disordered; but in our fourth case, which follows, the mental affection was one of the most distressing symptoms. The patient was reported to be a highly intelligent girl, nine years old; but when she first came under our care in April, 1836, the expression of her countenance was that of confirmed fatuity, and her actions and conversation were such as might have been expected from

* *Cimicifuga racemosa*, Nuttall, Torrey. *Cim. Serpentaria*, Pursh. *Actæa racemosa*, Willdenow. *Macrotys racemosa*, Eaton's Manual. *Vide* United States Dispensatory, by Wood and Bache.

her physiognomy. She walked with great difficulty, and appeared to have lost nearly all power over both her left extremities. She had irregularity of the bowels, headach, and often complained of pain shooting down the left arm. In this case, the symptoms had been gradually coming on for six months before we were consulted, but owing to domestic misfortunes, her widowed mother was able to give her much less attention than she deserved. Cups [cupping-glasses] were applied to the back of the head and neck, once; stimulating pediluvia were prescribed, and frictions with salt over the surface of the body. After being moderately purged, every day for a week, she commenced with the cimicifuga in decoction, rapidly increasing the dose. An improvement was evident from the first few days' use of this last remedy; her motions became less violent; control over her left side was gradually regained; the functions of the mind improved with her other symptoms, and at the end of four weeks from our first visit, to the astonishment of her friends and, we may add, to our own, she was entirely well.

By permission of our friend, Dr. Otto, we are allowed to refer to a very intractable case, which occurred in his own practice soon after the publication of Dr. Young's paper. A young lady of sixteen was reduced to an extreme state of prostration by the violence and constancy of her motions, which scarce allowed her to sleep at all during weeks; she had become completely idiotic, and a fatal termination was looked for. All the usual remedies had been employed, without advantage, when Dr. Otto directed the powdered black-snake root, as something still untried; and to his surprise an amendment took place, which resulted in a restoration to perfect health. The cure is attributed by her experienced and talented attendant entirely to the remedy last employed.

Another case, possessing much interest, occurred in the Pennsylvania hospital, in April, 1836, under the care of Professor Wood, then attending physician. This patient was a boy about seventeen years of age, from near Dyott's glass-works, suffering with a violent attack of chorea, and who, Dr. Wood informs us, was entirely cured by the remedy particularly under notice.

*American Journal of Med. Sciences,* No. 50. *Feb.* 1840.

---

*Smallpox in the Camel.* By J. W. WINCHESTER, M.D., of Jatta (India).

THE following facts connected with smallpox, and communicated to me by M. Masson, a gentleman well known for his very persevering, and highly successful antiquarian researches in central and upper Asia, will, I am sure, be interesting.

In the province of Lus, along the seacoast of Baloochistan, south-west of Karachi, of which Bhala is the capital and Somneanee the port, the milkers of camels affirm that they have a disease called "Photo Shootur." Smallpox in Lus is designated "Photo," so that the term "Photo Shootur" implies the smallpox of the camel, which is an eruption on the udder of that animal not more violent and in its pustule similar to that on the udder of the cow. The camels, while thus afflicted, continue to give milk, which is largely drank by the inhabitants, but both the men and the women who milk them are invariably seized with a pustular disease similar to that on the camel's udder, on their hands and arms, never extending above the elbows. No one has ever been known to die from this eruption, and the natives themselves remark, that those who have had the "Photo Shootur," are uniformly exempt from smallpox, which is a disease occasionally endemic in the district.

Although the inhabitants are aware that the "Photo Shootur" is a preventive against smallpox, they do not inoculate with its virus, in a manner similar to what they do from the smallpox pustule, which frequently brings on a disease, believed by these people to be beyond the power of the native doctors, in so much so, that the relatives of the sick proceed to the shrine of some favorite saint, there by propitiatory offerings to invoke aid in favour of the diseased.

*Indian Journal of Med. Science.* *Oct.* 1, 1839.

